# An Integrative Segmentation Framework for Cell Nucleus of Fluorescence Microscopy

**DOI:** 10.3390/genes13030431

**Published:** 2022-02-26

**Authors:** Weihao Pan, Zhe Liu, Weichen Song, Xuyang Zhen, Kai Yuan, Fei Xu, Guan Ning Lin

**Affiliations:** 1 School of Biomedical Engineering, Shanghai Jiao Tong University, Shanghai 200030, China; parrypwhppp@sjtu.edu.cn (W.P.); liuzlm1030@sjtu.edu.cn (Z.L.); goubegou@sjtu.edu.cn (W.S.); zhenxuyang@sjtu.edu.cn (X.Z.); yuankai2017@sibcb.ac.cn (K.Y.); 2 State Key Laboratory of Functional Materials for Informatics, Shanghai Institute of Microsystem and Information Technology (SIMIT), Chinese Academy of Sciences, Shanghai 200050, China; 3 College of Science, Donghua University, Shanghai 201620, China

**Keywords:** nucleus segmentation, deep learning, attention mechanism, fluorescence microscopy

## Abstract

Nucleus segmentation of fluorescence microscopy is a critical step in quantifying measurements in cell biology. Automatic and accurate nucleus segmentation has powerful applications in analyzing intrinsic characterization in nucleus morphology. However, existing methods have limited capacity to perform accurate segmentation in challenging samples, such as noisy images and clumped nuclei. In this paper, inspired by the idea of cascaded U-Net (or W-Net) and its remarkable performance improvement in medical image segmentation, we proposed a novel framework called Attention-enhanced Simplified W-Net (ASW-Net), in which a cascade-like structure with between-net connections was used. Results showed that this lightweight model could reach remarkable segmentation performance in the BBBC039 testing set (aggregated Jaccard index, 0.90). In addition, our proposed framework performed better than the state-of-the-art methods in terms of segmentation performance. Moreover, we further explored the effectiveness of our designed network by visualizing the deep features from the network. Notably, our proposed framework is open source.

## 1. Introduction

Image segmentation plays a vital role in cell biology in acquiring quantitative measurements [1]. From microscopy-based measurement [2], multiplex imaging analysis [3,4], to high-content screening [5,6], image segmentation is crucial to the characterization of cell signaling and morphology, such as the size and shape.

One of the critical steps for quantifying measurements in fluorescence microscopy is accurate nucleus segmentation. It is the first step for identifying cell borders [7], enabling each cell to characterize. Automatic nucleus segmentation alleviates the problems of assessing subtle visual features by manually inefficiently drawing the nuclear contours [8]. Over the past decades, some significant research efforts have been made to improve the performance of nucleus segmentation [9]. For instance, thresholding [10], watershed algorithm [11,12], and active contour [13,14] are some of the dominant approaches to this segmentation task. However, these methods highly rely on expert knowledge to set parameters, and adjustment parameters are required in different experimental conditions. Moreover, these classical algorithms do not work effectively in some challenging cases, such as noisy images and crowded nuclei [1,7,15].

Deep learning has recently succeeded in image segmentation tasks and often achieves a much higher performance on popular benchmarks than traditional methods [16]. From multi-organ segmentation on CT images [17,18] to tumor-tissue segmentation on histopathological images [19,20], deep learning has achieved outstanding performance improvement. These segmentation tasks can be formulated as a classification problem of each pixel with a semantic label [16]. Hence, nucleus segmentation can be considered as a problem of classifying each pixel into a semantic category. Some previous works illustrate that deep learning is effective in nucleus segmentation [1,21,22], such as U-Net [23] and W-Net [24]. The architecture of U-Net has been broadly thought of as an encoder convolutional neural network followed by a decoder network. The encoder extracts feature related to the segmentation task, and then the decoder constructs the mask given only such features. The traditional architecture of U-Net consists of basic convolution operation, pooling operation, and deconvolution operation. In contrast, W-Net is a network with cascaded U-shape architecture [24]. In W-Net, the between-net connections are designed to preserve features from shallow layers to deep layers by concatenation operations [24]. This cascade structure indicates a noticeable accuracy improvement. However, W-Net is a computationally heavy network due to its cascade architecture. Thus, a large amount of data would be required to train W-Net. Meanwhile, a longer time is required than U-Net for training a well-fitting model.

Given the importance of nucleus segmentation and only limited data for nucleus segmentation currently, in this study, we revisited the challenge of nucleus segmentation by incorporating the ideas from W-Net. In particular, inspired by U-Net and W-Net, we proposed a novel method for nucleus segmentation, named attention-enhanced simplified W-Net (ASW-Net). Our proposed model is a lightweight network utilizing a cascade-like structure with between-net connections. An attention gate is connected to the convolution block before each up-sampling process to extract representative features effectively. In this framework, we utilize the interior expansion, a post-processing method, to improve the nuclei segmentation accuracy.

In this study, we propose a novel framework with ASW-Net for the nucleus segmentation of fluorescence microscopy. Attention gates are applied to ASW-Net for better learning ability. Moreover, we visualize the deep features extracted from ASW-Net to interpret how this model effectively improves segmentation performance.

We have released ASW-Net as a publicly available segmentation tool for the community. The pre-trained model and all support materials are available at https://github.com/Liuzhe30/ASW-Net.

## 2. Materials and Methods

### 2.1. Benchmark Dataset

In total, we employed two image datasets in our experiment: the BBBC039 dataset [5] and a ganglioneuroblastoma image set [25].

BBBC039 is a high-throughput chemical screen on human osteosarcoma U2OS cell line, available from the Broad Bioimage Benchmark Collection (http://www.broad.mit.edu/bbbc) (accessed date: 22 May 2021). This image set only includes a channel of DNA of a single field of view. This collection has around 23,165 nuclei annotated by expert biologists, including variant perturbations from one experiment. The dataset consists of 200 images, each 520 × 696 pixels in size. We used the same data partitions as Broad Institute suggests comparing results with previous work. Hence, the collection is split into 100 training, 50 validation, and 50 testing images. Hence, more than 72 million pixels were used in our pixel classification task for nucleus segmentation.

The ganglioneuroblastoma dataset includes ten images/2773 nuclei. This image set is of broad heterogeneity regarding magnification (20×, 40×, or 63×). Moreover, this image set has a different signal-to-noise ratio (SNR). Overall, 70% of the dataset is normal SNR and 30% SNR data. We used this collection as external validation to check the generalization capability of ASW-Net in different magnification, different cell types, and different SNR.

### 2.2. Algorithm Framework

**Figure 1:**
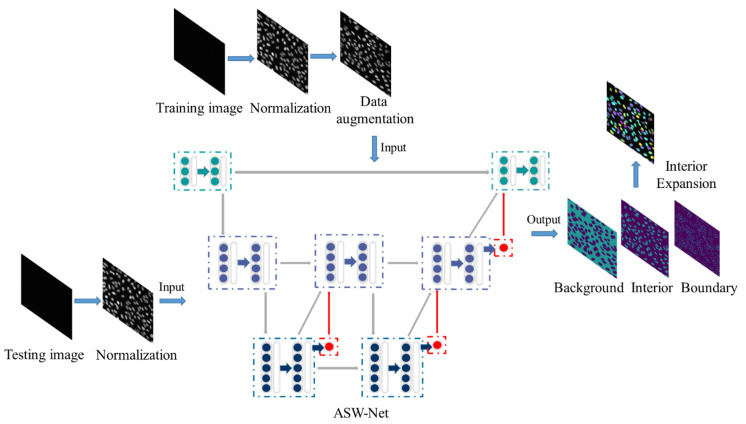
Overview of the proposed framework for nuclear instance segmentation. This pipeline contains image pre-processing, network architecture, and image post-processing. ASW-NET is trained based on the training images, and appropriate weights are found in the training process. In the testing process, the ASW-NET predicts the probability of each pixel’s background, interior, and boundary. Then an interior expansion algorithm is adopted to obtain the final instance segmentation results. The details of ASW-Net are explained in Figure 2.

Our overall framework for nuclear instance segmentation contains three parts: image pre-processing, network architecture, and image post-processing (see Figure 1). As for the pre-processing method, we first applied gray-scale normalization to make contours and instances clearer in the source image. To avoid as much over-fitting as possible, we also utilized some data augmentation operations for the training set (rotation and flip). Then, the processed images were fed into our proposed deep-learning network ASW-Net for training or testing to predict whether a given pixel is on the edge of the nucleus, inside the nucleus, or in the background. The detailed structure of ASW-Net is demonstrated in the next section. Considering the thick boundary in the original predicted results, we implemented the interior expansion algorithm to thin the boundary of nuclei and improve prediction accuracy. After the procedures described above, we obtained the final segmentation results.

### 2.3. Model Design

#### 2.3.1. Network Architecture

Inspired by the classical structures of U-Net [23], W-Net [24], and some pioneered research works, we proposed ASW-Net, a deep learning-based tool for cell nucleus segmentation of fluorescence microscopy. As a simplified W-net, ASW-Net can extract more features from raw images than U-net, and it is lighter than W-net at the same time. The attention mechanism also endows the model with better learning ability and interpretability.

**Figure 2:**
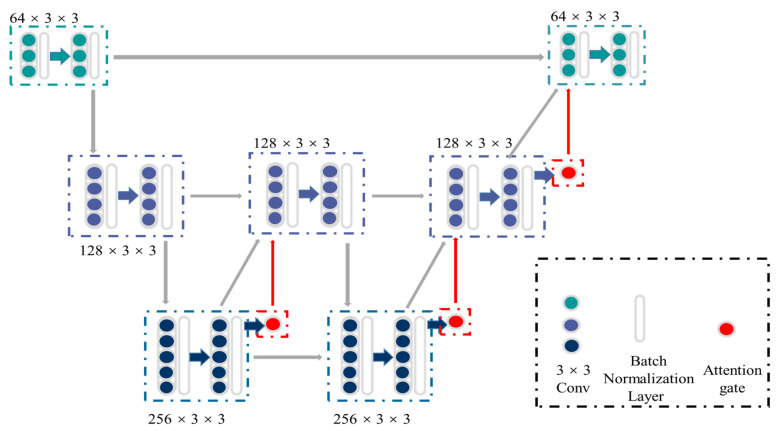
The structure of ASW-Net. There are three down-sampling processes (encoding phase), three up-sampling processes (decoding phase), and three attention gates, which form the shape of a ‘W’. The green and blue dots represent the convolutional blocks, while the white transparent boxes represent the batch normalization layers and the red dots represent the attention gates. The gray, green, blue, and red arrows represent the data flow.

Figure 2 shows the detailed structure of ASW-Net, which contains three down-sampling processes (encoding phase), three up-sampling processes (decoding phase), and three attention gates. First, the input images are fed into two successive down-sampling processes, followed by an up-sampling process. Since the original input consists of features from a shallow level and carries unusable noise with a high probability, the first up-sampling process does not feed it back into the model. To learn the deeper features more purely, we only decode and re-enter the information generated before the second down-sampling.

After this, we continued to conduct a down-sampling process followed by two up-sampling processes and combined the original input into the output information to merge the deep and shallow features. Besides, batch normalization layers were implemented to reduce saturation [26], and attention gates were attached to all the upper sampling blocks to increase the accuracy of predicting the boundary of objects. We then finally obtained the classification output by combining the original input with the up-sampled result and convolving them.

#### 2.3.2. Implementation Details

Our model was implemented, trained, and tested using the open-source software library Keras [27] and Tensorflow [28] on Nvidia Tesla v100 GPUs. The main hyperparameters, such as the kernel size of convolutional blocks and the learning rate, were explored. Besides, we also adopted an early stopping strategy and a save-best strategy. When the validation loss did not reduce in 10 epochs during training time, the training process would be stopped, and the best model parameters would be saved. In all cases, the weights were initialized by default setting in Keras; the parameters were trained using an RMSProp optimizer [29] to dynamically change the learning rate during model training. Our proposed network was trained by minimizing the weighted cross-entropy loss function [30] for semantic segmentation.

### 2.4. Post-Processing: Interior Expansion Algorithm to Convert a 3-Class Label to an Instance Label

We chose pixels with a probability of interior greater than 0.5 as seeds. For each seeded nucleus, it would expand its territory by one pixel at each iteration. During the iteration, the probability of the nucleus gradually declined, and the probability of the boundary increased. At last, the seeded nucleus stopped its expansion when the probability of the boundary reached the local maximum on its expansion direction. To avoid the seeded nucleus expanding to other nucleus regions in clumped objects, we discarded the boundary pixel in the last iteration. The union of these pixels and the pixels of the original interior seed were the ultimate output of this algorithm.

### 2.5. Performance Evaluation Metrics

A commonly used evaluation metric for nucleus segmentation is the Aggregated Jaccard Index (AJI). To quantitatively evaluate the performance of ASW-Net and other nucleus segmentation methods, we adopted four measures, including AJI [8], Dice coefficient (DICE1) [31], ensemble Dice (DICE2) [19], and panoptic quality (PQ) [21]. PQ is the product of Detection Quality (DQ) and Segmentation Quality (SQ). These metrics are used to compare the area of overlap between the predicted area of a nucleus and the ground-truth area of the nuclei. If we obtain a higher metric value, we will get more accurate downstream extracted metrics, such as area and perimeter.

## 3. Results

### 3.1. Performance of Proposed Framework and Other Existing Nucleus Segmentation Methods

To evaluate the prediction performance of our proposed method, we tested ASW-Net against CellProfiler (see Supplementary Note for parameter settings) [32], U-Net [23], and SW-Net (ASW-Net without attention gates) with ground truth as a baseline. Experimental results show that ASW-Net achieved satisfactory accuracy in classification even with insufficient labeled training samples (Figure S1). It can be seen in Table 1 that among all of the tested methods, our proposed framework has the highest score for all indicators except DICE2.

#### 3.1.1. ASW-Net Performs Better in Different Nuclear Density

**Figure 3:**
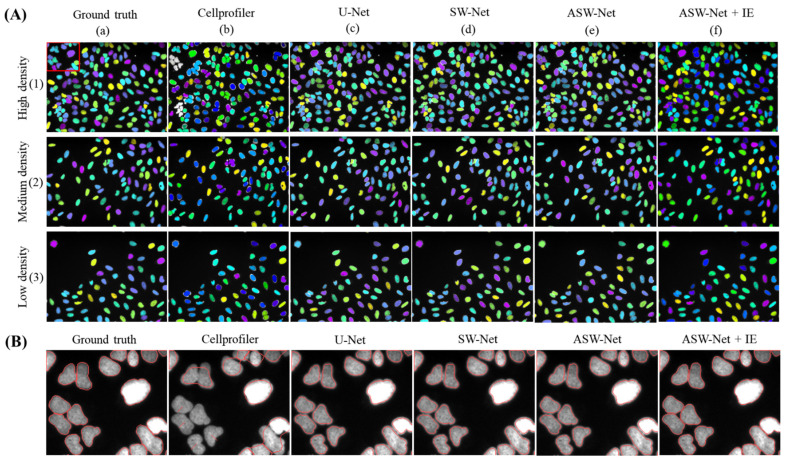
Visualized comparison between ASW-Net and other existing methods against the ground truth. (**A**) Three images with different nuclear distribution densities were randomly selected for this study. From top to bottom are high density, medium density, low density of nuclear images, respectively. From left to right are the masks of ground truth, the prediction of Cellprofiler, U-Net, SW-Net, ASW-Net, ASW-Net plus interior expansion, respectively. The nuclear masks are colored randomly. (**B**) Magnified segmentation result and the ground truth. From left to right are the mask outlines of ground truth, the prediction of Cellprofiler, U-Net, SW-Net, ASW-Net, ASW-Net plus interior expansion, respectively.

When it comes to the classification stability of ASW-Net, three images with different nuclear distribution densities were randomly selected for a case study. As shown in Figure 3A, CellProfiler performs poorly in the case of high nuclear density (see Figure 3A(b)(1)), while ASW-Net generates reliable masks no matter in the case of low, medium, or high nuclear density. Furthermore, SW-Net with the attention gates performs better than the network without the attention mechanism, proving the necessity of embedding the attention mechanism in our method.

#### 3.1.2. ASW-Net Excels at Segmentation in Low SNR Dataset

We tested our proposed framework on the ganglioneuroblastoma dataset, with a low signal-to-noise ratio and different cell types from the BBBC039 image set. We evaluated the performance of the framework on the high SNR and the low SNR ganglioneuroblastoma dataset, respectively. As shown in Table 2 and Table 3, none of the five methods performed as well as the previous dataset. The results confirm the difficulty in accurate nuclei segmentation. However, our proposed framework still achieved satisfactory segmentation results and outperformed other methods, especially in a low SNR ganglioneuroblastoma image set. It is worth mentioning that ASW-Net performs better than U-Net and SW-Net, the two deep learning networks without attention gates. It indicates that the attention mechanism used in ASW-Net probably helps the model transfer to this noisy dataset.

### 3.2. Ablation Study

To examine whether a particular component of ASW-Net was vital or necessary, we carried out an ablation study by removing some network elements. The experiments performed in this section shared the same features and hyper-parameters. As is shown in Table 4, the implementation of attention mechanisms, data augmentation (rotation and flip), and post-processing (watershed) all contribute to the proposed method. Specifically, the addition of image rotation and flip significantly improved the prediction performance (∆AJI = −1.451 for no rotation, ∆AJI = −1.214 for no flip), which may be due to the undiversified training dataset. Moreover, the data augmentation also helped improve the generalization capability of the model.

Attention gates were beneficial for achieving better prediction accuracy (∆AJI = −0.587 for no attention) at the same time. On the one hand, the introduction of attention gates made the number of parameters increase, thus enhancing the learning ability of the model. On the other hand, the attention gates attached in the up-sampling process were conducive to more efficient feature extraction.

We also tested whether two post-processing methods helped improve segmentation accuracy, including watershed and interior expansion algorithms. Both of these two methods have a positive effect on prediction performance. The introduction of the watershed improves the performance a little (∆AJI = +0.005), possibly because the watershed algorithm is helpful to separate the connected cell nucleus [33]. However, we do not observe a significant improvement in the segmentation performance by the watershed algorithm because ASW-Net could already predict boundary class effectively. Hence, the clumped nuclei would be separated well after thresholding on the probabilistic map of ASW-Net. Another post-processing method, interior expansion, was also proven effective by an ablation study. Compared with the thresholding method on a probabilistic map, the interior expansion algorithm automatically expanded its boundary to the most likely place and kept the final boundary only one pixel wide and continuous. Although our ASW-Net could predict the pixels into the background class, interior class, and boundary class with high accuracy, the performance could be further improved through post-processing methods.

### 3.3. Visualization of Deep Features Extracted from Images

**Figure 4:**
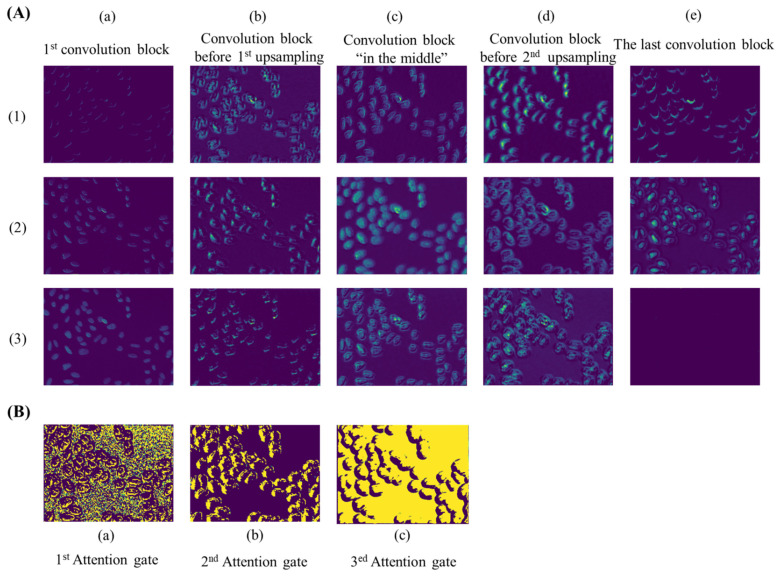
Visualization of feature maps extracted by convolution layers at different depths of ASW-Net. (**A**): (a) to (e), from left to right, represent the features extracted by the first convolution block, the convolution block before the first up-sampling, the convolution block in the middle, the convolution block before the second up-sampling, and the last convolution block, respectively. (**B**): (a) to (c), from left to right, represent the features extracted by the first, the second, and the third attention gate.

As an automatic feature extraction process, deep learning would learn high-level abstract features from original inputs [34]. Thus, to further explore the effectiveness of convolution blocks at different depths of ASW-Net, we visualized the feature map of each convolutional layer, and the results are shown in Figure 4A. As described earlier, the convolution block before the first up-sampling (Figure 4A(b)) and before the second up-sampling (Figure 4A(d)) incorporated the deepest abstract features, which means these two layers were able to capture more effective information for prediction. Specifically, the convolutional kernels presented by Figure 4A(b)(1), Figure 4A(d)(2), and Figure 4A(d)(3) all captured the edge of the cell nucleus and its adjacent pixels, which made the nuclear edge appear blurred and bolded in the corresponding feature maps. Figure 4A(b)(2), Figure 4A(b)(3), and Figure 4A(d)(1) show that these kernels played important roles in sharpening and positioning one side of the nucleus. Besides, it is evident that the feature map generated from the shallow convolution layer was much closer to the actual images through Figure 4A(a): the weight distribution of all feature maps looks very similar to the input images. Moreover, abstract features and raw information similar to input can both be seen in Figure 4A(c), since this layer combined the output of layers presented by Figure 4A(a) and Figure 4A(b). Finally, comparing the visualization results of the first convolution block (Figure 4A(a) with the last convolution block (Figure 4A(e), we observed that the final convolutional layer reflected the most favorable information for segmentation. The center of the nucleus and the edge of the nucleus are separated at the last layer (see Figure 4A(e)(2)), which proves the effectiveness and interpretability of the deep neural network.

As for attention gates (see Figure 4B), with the deepening of the network, the distribution of weight gradually changes from distraction to focus on edge information. This indicates that attention gates had learned which parts were worthy of attention, which is undoubtedly helpful for the improvement of model prediction ability.

### 3.4. Strong Correlation of Downstream Metric Derived from Experts and Proposed Framework

**Figure 5:**
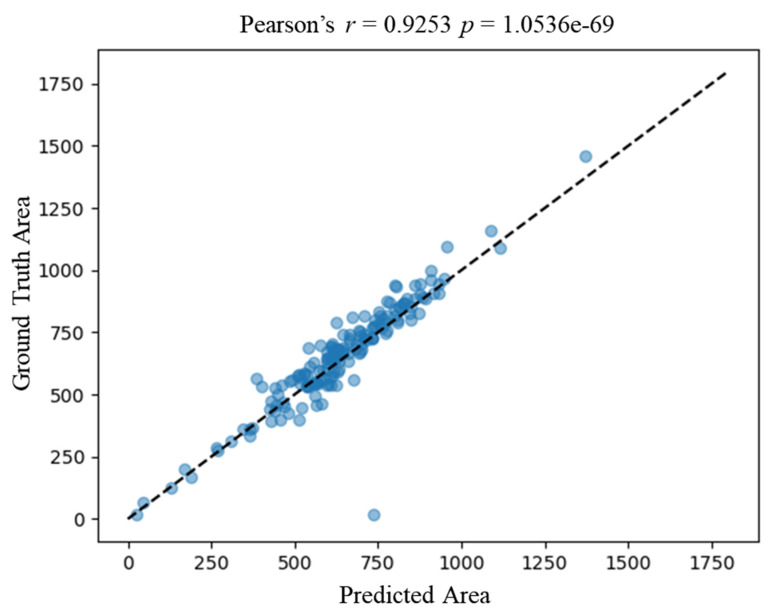
Correlation between predicted area by the proposed framework and the ground truth area. The *x*-axis represents the predicted area of an image, and the *y*-axis represents the ground truth area. Each blue dot represents a nucleus. The dashed line represents the ideal correlation. Area_ground_truth = Area_predicted.

We verified the correlation of downstream metrics derived from the ground truth annotation by experts and our proposed framework. We then calculated the area of each nucleus derived from expert annotation and automatic segmentation by the proposed framework. Figure 5 shows a strong correlation of the nucleus area between the ground truth and the prediction, with a correlation coefficient of 0.9253 (Pearson *p*-value < 0.05). The correlation result verifies the accuracy of automatic segmentation at the cellular level.

## 4. Discussion

Nucleus segmentation is a prerequisite for automated downstream analysis, including characterization of nucleus morphology, identification and detection of cells, and quantitative measurement of protein expression. However, manually drawing the nuclear contours is time-consuming and laborious. Automatic and accurate nucleus segmentation would alleviate this difficulty and enable biologists to yield insights into intrinsic features of nucleus morphology.

In this study, we proposed a deep learning-based framework, which is based on ASW-Net, to automatically segment the nucleus of fluorescent microscopy by integrating advantages of attention mechanism, W-Net, and adopting a cascade-like structure with between-net connections. We have shown that our lightweight model outperforms the-state-of-art methods in segmentation performance. We also showed that utilizing the attention mechanism would provide information about nucleus segmentation, leading to a much-improved performance. The attention mechanism endows our proposed tool with better learning ability and interpretability, making our tool likely to translate well to practical usage. The automatic segmentation results by our proposed framework are consistent with the ground truth, which is verified by the downstream metric, such as the area of each nucleus. We believe that the automatic segmentation framework can mitigate the difficulty of drawing nucleus contours manually and provide more accurate segmentation than other tools.

While our proposed tool achieves a promising performance in nucleus segmentation of fluorescent microscopy, there are a couple of technical limitations from the aspect of deep learning techniques, such as a theoretical explanation of our architecture effectiveness and tuning hyper-parameters for an optimal model. We also compared our proposed framework with the Cellpose architecture [35]. Our proposed framework outperformed the Cellpose architecture on the BBBC039 dataset and performed about 3% worse than the Cellpose architecture on the ganglioneuroblastoma dataset, which is expected since the number of parameters used for training by Cellpose is much greater than our ASW-Net. At the same time, our lightweight ASW-Net could produce the result faster. In summation, the introduction of attention mechanism and the resolution fusion module in ASW-Net can also be applied in other backbones of neural networks and help improve performance.

## 5. Conclusions

We implemented ASW-Net as a publicly available segmentation tool for the research community. The model achieves remarkable performance in nucleus segmentation. In the future, our model can be retrained on other types of nucleus images, such as highly multiplexed imaging data.

## Figures and Tables

**Table 1:** The performance comparison between ASW-Net and other existing methods on BBBC039 nuclei set.

Method	DICE1	DICE2	AJI	DQ	SQ	PQ
CellProfiler [32]	87.884	73.491	67.740	81.165	79.319	64.506
U-Net [1]	89.094	86.916	78.717	92.395	79.562	73.646
SW-Net	89.282	87.466	79.219	91.666	80.043	73.505
ASW-Net	89.642	87.518	79.806	91.666	80.627	74.058
ASW-Net + Interior expansion	96.452	84.798	90.200	94.431	91.670	86.645

**Table 2:** The performance comparison on high SNR ganglioneuroblastoma dataset.

Method	DICE1	DICE2	AJI	DQ	SQ	PQ
CellProfiler [32]	0.64476	0.46786	0.29697	0.31771	0.64703	0.20601
U-Net [1]	0.78672	0.74143	0.57064	0.71474	0.69285	0.49442
SW-Net	0.78661	0.74262	0.56630	0.70997	0.69462	0.49268
ASW-Net	0.78957	0.74885	0.56910	0.73218	0.70059	0.51276
ASW-Net + Interior expansion	0.84228	0.72494	0.61944	0.76275	0.79579	0.60729

**Table 3:** The performance comparison on low SNR ganglioneuroblastoma dataset.

Method	DICE1	DICE2	AJI	DQ	SQ	PQ
CellProfiler [32]	0.59226	0.61923	0.35380	0.44781	0.69943	0.31294
U-Net [1]	0.49404	0.52160	0.30319	0.35329	0.61444	0.21976
SW-Net	0.48451	0.53914	0.30149	0.32245	0.61859	0.20240
ASW-Net	0.61887	0.60938	0.39892	0.50748	0.66386	0.33850
ASW-Net + Interior expansion	0.65787	0.60340	0.42803	0.54613	0.71036	0.38913

**Table 4:** Ablation study of ASW-Net.

Aspect	System Variant	AJI	ΔAJI
	ASW-Net	79.806	-
Attention	No Attention	79.219	−0.587
Augmentation	No rotation	78.355	−1.451
	No flip	78.592	−1.214
Post-processing	Watershed	79.811	+0.005
	Interior expansion	90.200	+10.394

## Data Availability

All data analyzed in this study are curated from public domain.

## Supplementary Materials

The following are available online at https://www.mdpi.com/article/10.3390/genes13030431/s1, Figure S1: Receiver operating characteristic of pixel classification of ASW-Net. Blue, orange, green, and red curves are average background, interior, and boundary ROC curves, respectively. Supplementary Note: Cellprofiler pipeline parameter settings.
