# Supplementary material for: An Integrative Segmentation Framework for Cell Nucleus of Fluorescence Microscopy
Source: Genes (Basel). 2022 Feb 26;13(3):431. doi: 10.3390/genes13030431 (PMC8950038; doi:10.3390/genes13030431)
Supplement: Supplementary file 1 [file genes-13-00431-s001.zip › genes-1579844-supplementary.pdf]

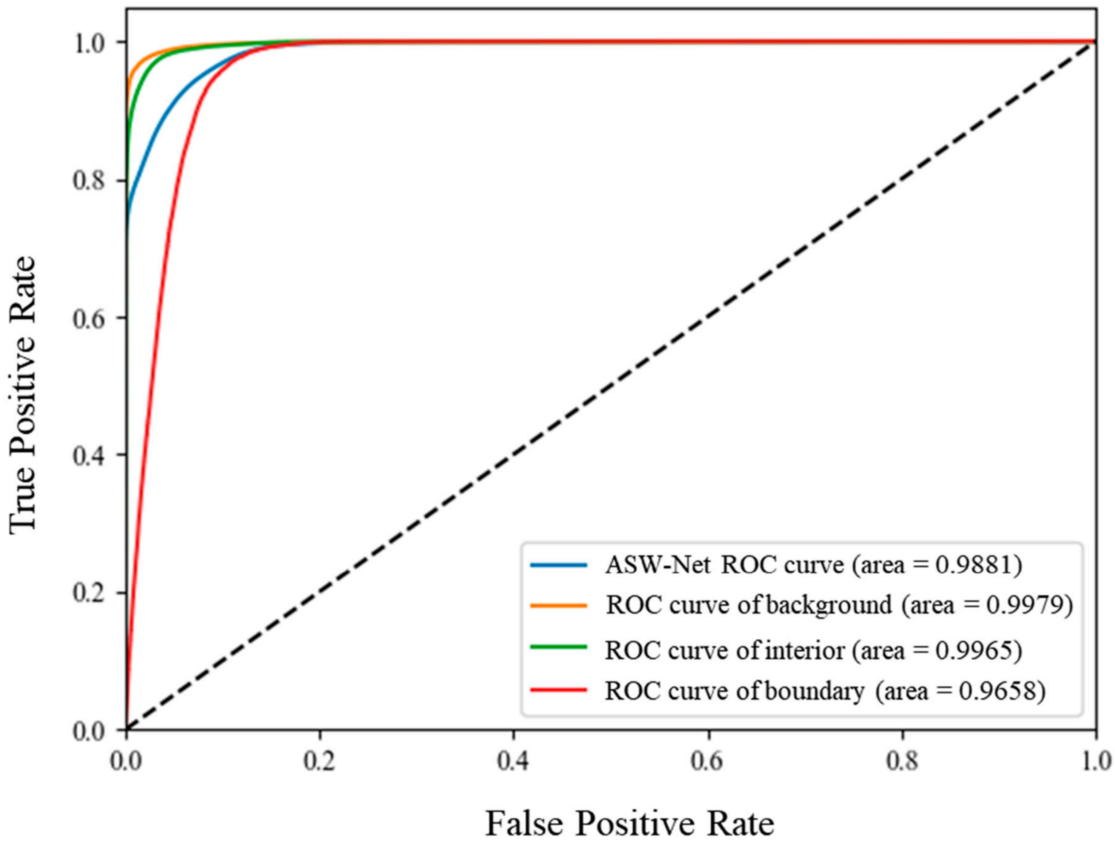

Figure S1. Receiver operating characteristic of pixel classification of ASW-Net. Blue, orange, green, and red curves are average background, interior, and boundary ROC curves, respectively.

Supplementary Note - Materials and Methods

Cellprofiler pipeline parameter settings

We developed a pipeline for configuring parameters in Cellprofiler. The pipeline mainly included the Smooth module and IdentifyPrimaryObjects module. We first utilized the smooth module for the Gaussian blur algorithm for noise reduction. Next, we used the IdentifyPrimaryObjects module for nuclei segmentation. We applied the Otsu threshold strategy to discriminate the foreground and the background of the image. Then we chose the best parameter settings to distinguish clumped objects after many attempts. We finally chose the intensity method to distinguish clumped objects according to the shape method to draw dividing lines between clumped objects.
